# Together JUN and DDIT3 (CHOP) control retinal ganglion cell death after axonal injury

**DOI:** 10.1186/s13024-017-0214-8

**Published:** 2017-10-02

**Authors:** Stephanie B. Syc-Mazurek, Kimberly A. Fernandes, Michael P. Wilson, Peter Shrager, Richard T. Libby

**Affiliations:** 1 Department of Ophthalmology, Flaum Eye Institute, University of Rochester Medical Center, Box 314, 601 Elmwood Ave, Rochester, NY 14642 USA; 2 Neuroscience Graduate Program, Rochester, USA; 3 Department of Neuroscience, Rochester, USA; 4 Department of Biomedical Genetics, Rochester, USA; 5 The Center for Visual Sciences, University of Rochester Medical Center, Rochester, NY 14642 USA

**Keywords:** Axonopathy, Neurodegeneration, Retinal ganglion cell, Mitogen-activated protein kinase, Endoplasmic reticulum stress

## Abstract

**Background:**

Optic nerve injury is an important pathological component in neurodegenerative diseases such as traumatic optic neuropathies and glaucoma. The molecular signaling pathway(s) critical for retinal ganglion cell (RGC) death after axonal insult, however, is/are not fully defined. RGC death after axonal injury is known to occur by BAX-dependent apoptosis. Two transcription factors JUN (the canonical target of JNK) and DDIT3 (CHOP; a key mediator of the endoplasmic reticulum stress response) are known to be important apoptotic signaling molecules after axonal injury, including in RGCs. However, neither *Jun* nor *Ddit3* deficiency provide complete protection to RGCs after injury. Since *Jun* and *Ddit3* are important apoptotic signaling molecules, we sought to determine if their combined deficiency might provide additive protection to RGCs after axonal injury.

**Methods:**

To determine if DDIT3 regulated the expression of JUN after an axonal insult, mice deficient for *Ddit3* were examined after optic nerve crush (ONC). In order to critically test the importance of these genes in RGC death after axonal injury, RGC survival was assessed at multiple time-points after ONC (14, 35, 60, and 120 days after injury) in *Jun*, *Ddit3,* and combined *Jun/Ddit3* deficient mice. Finally, to directly assess the role of JUN and DDIT3 in axonal degeneration, compound actions potentials were recorded from *Jun*, *Ddit3,* and *Jun*/*Ddit3* deficient mice after ONC.

**Results:**

Single and combined deficiency of *Jun* and *Ddit3* did not appear to alter gross retinal morphology. *Ddit3* deficiency did not alter expression of JUN after axonal injury. Deletion of both *Jun* and *Ddit3* provided significantly greater long-term protection to RGCs as compared to *Jun* or *Ddit3* deficiency alone. Finally, despite the profound protection to RGC somas provided by the deficiency of *Jun* plus *Ddit3*, their combined loss did not lessen axonal degeneration.

**Conclusions:**

These results suggest JUN and DDIT3 are independently regulated pro-death signaling molecules in RGCs and together account for the vast majority of apoptotic signaling in RGCs after axonal injury. Thus, JUN and DDIT3 may represent key molecular hubs that integrate upstream signaling events triggered by axonal injury with downstream transcriptional events that ultimately culminate in RGC apoptosis.

## Background

Axonal injury is an important component in neurodegenerative diseases, including traumatic optic neuropathies and glaucoma [1–4]. In glaucomatous neurodegeneration, axonal injury at the lamina of the optic nerve head leads to axonal dysfunction and apoptotic RGC death [2, 4–20]. Several pro-death molecular signaling pathways have been implicated in glaucomatous RGC death including mitogen-activated protein kinase (MAPK) and endoplasmic reticulum (ER) signaling [21–37]. The molecular signaling leading from ocular hypertensive insult to apoptotic RGC death, however, is not well defined. Identifying the molecular events leading to RGC apoptosis after a glaucoma-relevant insult is an important step in identifying the molecules responsible for triggering and signaling axonal and somal degeneration in glaucoma.

MAPK pathway components, including c-Jun N-terminal kinases (JNKs) and their canonical target JUN (also known as cJUN), and ER stress signaling, and its downstream effector DNA damage inducible transcript 3, *Ddit3* (also known as CCAAT/enhancer binding homologous protein (CHOP)/GADD153), are known to be important pro-apoptotic cascades after glaucoma-relevant injuries [21–36]. Activation of either JUN or DDIT3 is known to culminate in BAX activation and *Bax*-dependent apoptosis and BAX is known to be required for RGC death after axonal injury [6, 38, 39]. Importantly, either *Jun* or *Ddit3* deficiency has been shown to protect RGCs after axonal injury. Either *Jun* or *Ddit3* deficiency provided significant, but incomplete protection to RGCs after axonal injury [22, 33]. These data suggest that individually, *Jun* and *Ddit3* are not solely responsible for BAX activation in RGCs after axonal injury. It is possible that JUN and DDIT3 are functioning in the same pathway, and thus protecting RGCs through similar downstream targets. In other systems, ER stress can lead to JNK-JUN activation [40–42]. Furthermore, JUN and DDIT3 may be activated in RGCs by the same upstream kinase, dual leucine kinase (DLK) [35, 36, 43]. Alternatively, JUN and DDIT3 may be independently regulated after axonal injury. Therefore, it must be determined if JUN and DDIT3 act through distinct mechanisms in RGCs and provide additive protection to RGCs after axonal injury.

Here, we sought to evaluate if JUN and DDIT3 independently regulate RGC death after axonal injury or if they might function in the same molecular pathway. Previously we showed that DDIT3 upregulation after axonal injury was not prevented by *Jun* deficiency [23]. We extend these findings and show that similarly, *Ddit3* does not control expression of JUN [23]. Thus, JUN and DDIT3 appear to be independently regulated, suggesting that combined deficiency of both JUN and DDIT3 could provide greater protection than either deficiency alone. To determine if protection afforded by JUN and DDIT3 deficiency is additive, animals deficient in both *Jun* and *Ddit3* were generated and evaluated after mechanical optic nerve injury. Combined deficiency o*f Jun* and *Ddit3* was more protective of RGCs after axonal injury than either *Jun* or *Ddit3* deficiency alone, and provided profound long-term protection of RGCs after axonal injury. These results suggest that MAPK and ER stress signaling pathways are together the major regulators of RGCs after axonal injury.

## Methods

### Mice

Mice were housed on a 12-h light dark cycle and fed chow and water ad libitum. Five different alleles were used to generate four different mice strains: 1) mice deficient in *Jun* (also known as *cJun*), 2) mice deficient in *Ddit3*, 3) mice deficient in both *Jun* and *Ddit3*, and 4) mice deficient in *Dlk*. To generate animals conditionally deficient in *Jun,* a floxed allele of *Jun* [44] and Six3*-*cre recombinase (a neural retina cre, Jackson Laboratory, Stock# 019755) were crossed to generate 1) animals carrying the recombined floxed alleles, referred to as *Jun* ^−/−^ or *Jun* deficient (*Jun* ^fl/fl^Six3cre^+^), 2) heterozygote animals referred to as *Jun* ^+/−^(*Jun* ^*+*/fl^Six3cre^+^), and 3) animals carrying non-recombined floxed alleles or wildtype alleles with or without the cre recombinase referred to as *Jun* ^+/+^ or wildtype (WT, *Jun* ^*+/+*^Six3cre^*−*^, *Jun* ^*+/+*^Six3cre^*+*^, *Jun* ^*+/fl*^Six3cre^*−*^, or *Jun* ^*fl/fl*^ Six3cre^*−*^). Mice carrying the null allele for *Ddit3* were acquired from Jackson Laboratory (Stock# 005530) and intercrossed to generate: 1) animals carrying two copies of the null allele, referred to as *Ddit3* ^−/−^ or *Ddit3* deficient, 2) heterozygote animals carrying one copy of the null allele referred to as *Ddit3* ^−/+^, and 3) animals carrying no copies of the null allele, referred to as *Ddit3* ^+/+^ or WT in the text. Animals deficient in both *Jun* and *Ddit3* were generated by crossing animals carrying the floxed allele of *Jun* and Six3-cre with animals carrying the null allele for *Ddit3* to generate Ddit3^−/−^;*Jun* ^fl/fl^Six3cre^+^ animals referred to as *Jun/Ddit3* deficient animals in the text. To generate animals conditionally deficient in *Dlk,* a floxed allele of *Dlk* [45] and Six3*-*cre recombinase (a neural retina cre) were crossed on a C57BL/6 J background to generate 1) animals carrying the recombined floxed alleles, referred to as *Dlk* ^−/−^ or *Dlk* deficient (*Dlk* ^f/fl^Six3cre^+^) and 2) animals carrying non-recombined floxed alleles or wildtype alleles with or without the cre recombinase referred to as *Dlk* ^+/+^ or WT (*Dlk* ^*+/+*^Six3cre^*−*^, *Dlk* ^*+/+*^Six3cre^*+*^, *Dlk* ^*+/fl*^Six3cre^*−*^, or *Dlk* ^*fl/fl*^ Six3cre^*−*^). The *Jun* ^*fl*^ and *Dlk* ^*fl*^ alleles were all backcrossed at least 3 times to the C57BL/6 J genetic background prior to intercrossing with the Six3cre allele that had been backcrossed at least 10 generations into the C57BL/6 J genetic background. The *Ddit3* allele was obtained already backcrossed into C57BL/6 J for 5 generations. All experiments were conducted in adherence with the Association for Research in Vision and Ophthalmology’s statement on the use of animals in ophthalmic and vision research and were approved by the University of Rochester’s University Committee on Animal Resources.

### Optic nerve injury

Mice were anesthetized with 100 mg/kg ketamine and 10 mg/kg xylazine. Optic nerve crush (ONC) was completed as previously described [22, 23, 43, 46]. Briefly, the optic nerve was surgically exposed and crushed immediately behind the eye with a pair of self-closing forceps (Roboz RS-5027) for 5 s. Controls included contralateral eyes that had not been manipulated and eyes in which a sham surgery was performed, where the optic nerve was exposed but not crushed. Animals were harvested at 1, 5, 14, 35, 60, and 120 days following ONC or sham surgery.

### Histology and cell counts

Eyes to be processed for retinal morphology were fixed in 2.5% glutaraldehyde, 2% paraformaldehyde (PFA) for 24 h at 4 °C. After dehydration, eyes were embedded in Technovit (Electron Microscopy Services) and sectioned at 2.5 μm. Sections that included the optic nerve were stained with Multiple Stain Solution (Polysciences, Inc). Eyes to be processed for retinal flat mount immunohistochemistry were fixed in 4% PFA for two hours at room temperature. The anterior segment was removed and the posterior segment was processed for retinal flat mount immunohistochemistry as has been previously described [22, 23, 43, 46].

For retinal flat mount immunohistochemistry, retinas were dissected free of the optic cup and were blocked in 10% horse serum in 0.3% TritonX in 1 M PBS (phosphate buffered saline) for 24 h at 4 °C and then incubated in primary antibody for 72 h at 4 °C. Primary antibodies included: rabbit anti-JUN (Abcam, 1:250), rabbit anti-pJUN (Cell Signaling, 1:250) and mouse βIII tubulin (TUJ1; Covance, 1:1000). Whole retinas were washed in PBS and then incubated in secondary antibody, Alexafluor-conjugated secondary antibodies (Invitrogen), for 48 h at 4 °C before being washed in PBS and mounted RGC side up with Fluorogel in TRIS buffer (Electron Microscopy Sciences). Eight 40× fields were obtained for quantification of TUJ1+ and JUN+ cells. Images were taken 220 μm from the edge of the retina and equally spaced around the periphery of the retina (two fields per quadrant) as RGC density is known to vary across different dorso-ventral/medial-lateral retinal quadrants. Quantification was completed using the cell-counter tool in ImageJ.

### Western blotting

Mouse retinas were dissected and placed in 100 μl ice cold lysis buffer (1X RIPA buffer (Santa Cruz 24,948) containing 50 μM sodium fluoride, 2 mM PMSF, 2 mM sodium orthovanadate, 1X protease/phosphatase inhibitor cocktail (Cell Signaling 5872S)). Tissue was lysed by sonication (Bransa Digital Sonifier, 10% amplitude for 3 s) prior to spinning down cellular debris in a microcentrifuge (10,000 rotations per minute, 4°C, 5 min). 10 μl of supernatant was combined with 10 μl 2X Laemmli loading buffer (Bio-Rad) and boiled for 10 min. Samples were fractionated by SDS-PAGE on a 12% gel and transferred to a PVDF membrane (transfer buffer: 1X Tris-glycine (Bio-Rad), 20% methanol). Membranes were rinsed with double-distilled H_2_O and treated with a Qentix Western Blot Signal Enhancer kit (Thermo Scientific 21,050) according to manufacturer’s instructions. Membranes were then blocked at room temperature using 5% milk in TBST buffer for 1 h. Membranes were treated overnight at 4°C with one of the following primary antibodies: rabbit anti-JUN (Cell Signaling 9165S, 1:750) or mouse anti-GAPDH (Calbiochem CB1001, 1:2000). The following day membranes were washed 3X with TBST prior to treatment with secondary antibodies: HRP-conjugated anti-rabbit IgG (Bio-Rad 170–6515, 1:5000) or HRP-conjugated anti-mouse IgG (Bio-Rad 170–6516, 1:5000). Immunoreactive bands were detected using a chemiluminescence kit (Immun-star, Bio-Rad 170–5070) prior to exposure using either film or digital detection equipment (Azure Biosystems c500). Membranes were occasionally stripped following development and treated with another primary antibody (stripping buffer: 0.1 M Tris-Cl pH 6.8, 2% SDS, 0.7% β-mercaptoethanol). Densitometric analysis was conducted using ImageJ software, and pixel densities of experimental bands were normalized to those of GAPDH loading controls.

### Electrophysiology

Optic nerve compound action potentials (CAPs) were recorded as described previously [43]. Briefly, animals were euthanized with CO_2_, and optic nerves were dissected by transecting close to the eye and to the chiasm. Optic nerves were incubated for a minimum of 60 min in artificial cerebrospinal fluid (ACSF) aerated with 95% oxygen/5% CO_2_ at room temperature. ACSF was prepared with (in mM): 125 NaCl, 1.25 NaH_2_PO_4_, 25 glucose, 25 NaHCO_3_, 2.5 CaCl_2_, 1.3 MgCl_2_, and 2.5 KCl. The recording chamber was temperature controlled and perfused with oxygenated ACSF. The nerve was gently drawn into suction pipette electrodes containing ACSF at both the stimulating (retinal) end and the recording (chiasm) end. Stimuli consisted of supramaximal constant current pulses of 50 microseconds duration. CAP amplitudes were normalized by maintaining the ratio of the recording pipet plus nerve resistance to the recording pipet resistance at 1.7 [43]. All traces were taken at 25 °C. Data were digitized and then analyzed off-line.

### Statistical analysis

At least four retinas were analyzed for each genotype for all experimental conditions. The experimenters were masked to genotype and experimental cohort for quantification of cell number. The student’s t-test (unpaired) was used to compare differences across two groups. One-way ANOVA followed by the Tukey’s post hoc test for multiple comparisons was used for experiments comparing differences at a single time across more than two groups. Statistical significance was considered for *P* values <0.05.

## Results

### *Jun* and *Ddit3* deficiency does not alter retinal morphology

**Fig. 1:**
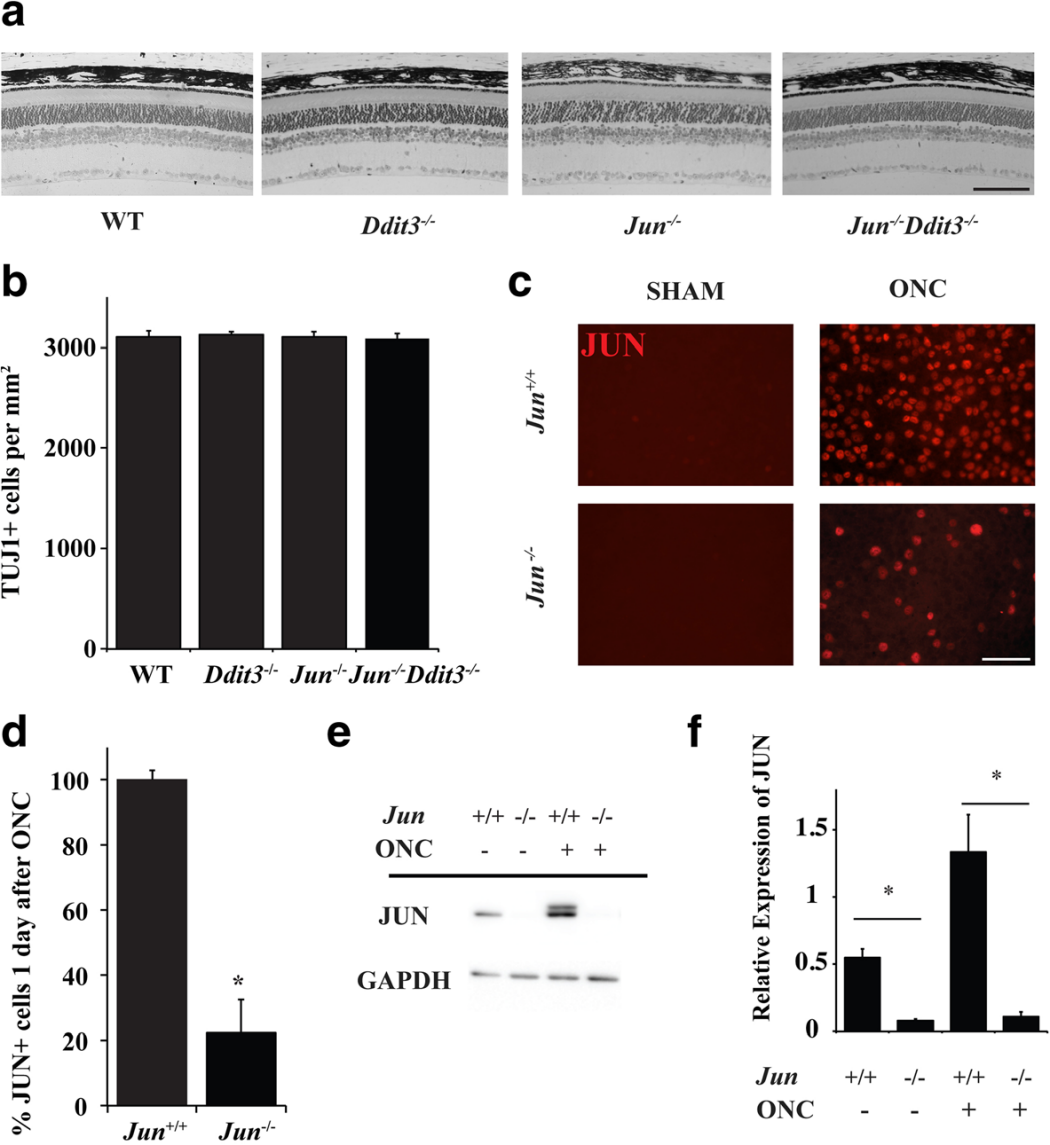
*Jun* and *Ddit3* deficiency does not alter retinal morphology. **a** Semi-thin retinal cross sections were taken to evaluate the gross structure of the retina in WT, *Jun*, *Ddit3,* and *Jun/Ddit3* deficient animals. *Jun*, *Ddit3,* and combined *Jun/Ddit3* deficiency did not appear to alter gross retinal structure. **b** TUJ1+ cells (a marker of RGCs) were counted in retinal flat mounts in WT, *Jun*, *Ddit3, Jun/Ddit3* deficient animals. No difference was observed across genotypes (*P* > 0.05 for each comparison; *n* = 8 per group). **c/d** JUN accumulation was readily detected in the somas of RGCs of wildtype animals after ONC but was not easily detected in sham retinas. To determine the efficiency of *Jun* recombination using Six3-cre, the number of JUN+ cells were counted 1 day after controlled optic nerve ONC, a time when JUN is widely expressed prior to RGC death. JUN+ cells are reduced by 77.7% in *Jun* deficient retinas after ONC (error bars represent standard error of the mean; *n* = 6 per genotype; *P* < 0.001; scale bar 50 μm). **e/f** Western blot analysis was used to determine the level of JUN in wildtype (+/+) and *Jun* deficient (−/−) animals. JUN was significantly reduced in both *Jun* deficient unmanipulated retinas (85%, ONC -) and *Jun* deficient retinas after ONC (92%, ONH +) as compared to wild type animals (* *p* < 0.01)

To determine if *Jun* (also known as *cJun*) and/or *Ddit3* (which encodes the protein CHOP) is necessary for retinal development; adult retinas deficient in *Jun, Ddit3*, and combined *Jun/Ddit3* were evaluated. *Jun* was conditionally deleted in the retina using Six3-cre, an established cre recombinase which deletes floxed alleles in the early developing neuroepithelium [47]. Animals carrying the null allele for *Ddit3* were evaluated independently and also crossed with animals carrying the *Jun* floxed allele and Six3-cre to generate animals deficient in both *Jun* and *Ddit3*. Deficiency in either *Jun* or *Ddit3* or the combined deficiency of *Jun* and *Ddit3* did not alter morphology of the retina (Fig. 1A). Furthermore, no differences were observed across subgroups in the number of TUJ1+ RGCs in adult animals (Fig. 1B; *P* > 0.05; *n* = 8 per group). Thus, neither *Jun* nor *Ddit3* deficiency appears to alter the normal development of the retina. To assess the recombination efficiency of Six3-cre, the number of RGCs expressing JUN was evaluated one day after ONC. Consistent with previous studies, JUN accumulation was readily detected in the somas of RGCs of wildtype animals after axonal injury but was not easily detected in sham retinas (Fig. 1C), [22, 23, 48]. In retinas with conditional deletion of *Jun,* JUN accumulation after ONC was detected in approximately 22% of RGCs after ONC compared to wildtype retinas (Fig. 1D, *P*<0.001). Western blot analysis was completed to assess the level of JUN in wildtype and *Jun* deficient animals (Fig. E and F). In unmanipulated eyes, JUN was reduced 85% (*p* < 0.001) in *Jun* deficient retinas as compared to wildtype retinas. After ONC, JUN was reduced 92% (*p* = 0.004) in *Jun* deficient eyes as compared to wildtype animals. Thus, while recombination was not complete, *Jun*-mediated changes should be greatly reduced in *Jun* deficient conditional mutants.

### *Jun* and *Ddit3* are independently regulated after optic nerve injury

**Fig. 2:**
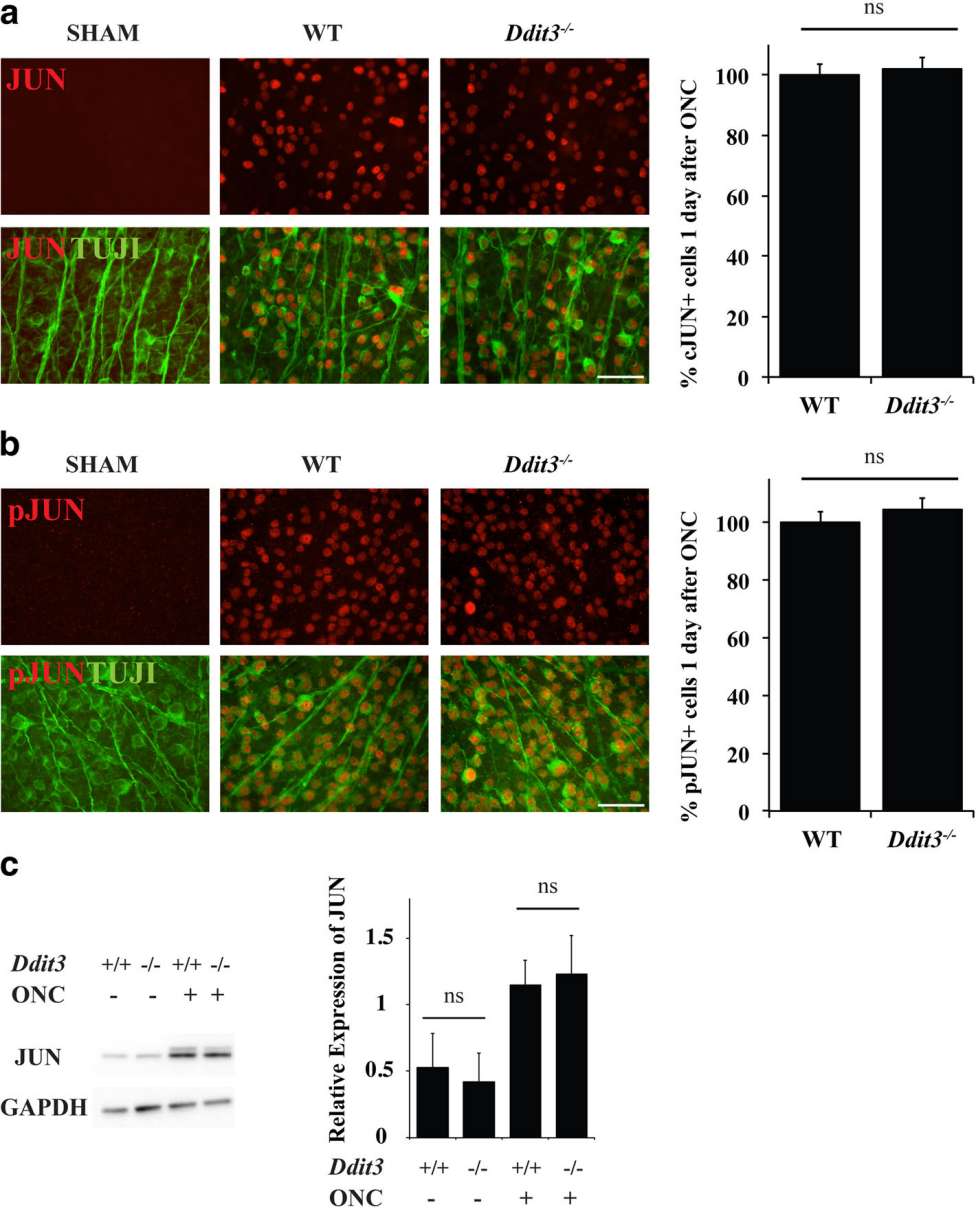
*Jun* and *Ddit3* are independently regulated after optic nerve injury. To determine if *Ddit3* regulates JUN and pJUN expression, retinal flat mounts (RGC side up) were examined from WT and *Ddit3* deficient retinas one day after ONC. **a** JUN (red) accumulates in RGCs (labeled with TUJ1, a marker of RGCs, green) in WT and *Ddit3* deficient retinas one day after ONC, but not in sham retinas (*n* = 4 per genotype and experimental condition). There was no significance difference (ns) in the number of JUN positive RGCs between wildtype and *Ddit3* deficient retinas. **b** Similarly, pJUN (red) accumulates in RGCs (green) in WT and *Ddit3* deficient retinas one day after ONC. There was no significance difference (ns) in the number of pJUN positive RGCs between wildtype and *Ddit3* deficient retinas. **c** Western blot analysis was used to determine the level of JUN in wildtype (+/+) and *Ddit3* deficient (−/−) animals. There was no significant difference in JUN levels between *Ddit3* deficient and wildtype unmanipulated eyes (ONC -) or between *Ddit3* deficient and wildtype eyes after ONC (ONC +). Previously, DDIT3 has been shown to be expressed in *Jun* deficient retinas after ONC [23]. Scale bar: 50 μm

Previously, JUN has been shown to be an important pro-apoptotic signaling pathway after glaucoma-relevant axon injury [21–26, 28–30, 35, 36]. DDIT3 has also been shown to be expressed after axonal insult [23, 31, 33, 34]. Furthermore, there is evidence to suggest that DDIT3 and JUN may act in the same signaling pathway. Accumulation of the DDIT3-dependent micro-RNA, miR-216b, has been found to directly target JUN [49]. Previously, *Jun* deficiency was found to not prevent the upregulation of DDIT3 protein after crush [23]. To determine if JUN is regulated by *Ddit3*, immunohistochemistry was performed on wildtype and *Ddit3* deficient retinas. JUN accumulated in RGC somas after optic nerve injury (ONC) in both WT and *Ddit3* deficient retinas (Fig. 2A). Quantification of JUN positive RGCs demonstrated no significant difference between JUN positive RGCs in wildtype and *Ddit3* deficient retinas. Likewise, quantification of phosphorylated JUN positive RGCs demonstrated no significance difference between wildtype and *Ddit3* deficient retinas after ONC (Fig. 2B). Western blot analysis was then completed both in unmanipulated eyes and after ONC to quantify the levels of JUN protein in wildtype and *Ddit3* deficient retinas. No significant difference in JUN levels was observed between wildtype and *Ddit3* deficient retinas in either condition. Together these results suggest that JUN expression is not dependent on *Ddit3* and that *Jun* and *Ddit3* may be independently regulated after optic nerve injury.

### Combined deficiency of *Jun* and *Ddit3* is more protective after axonal injury than either *Jun* or *Ddit3* deficiency alone

**Fig. 3:**
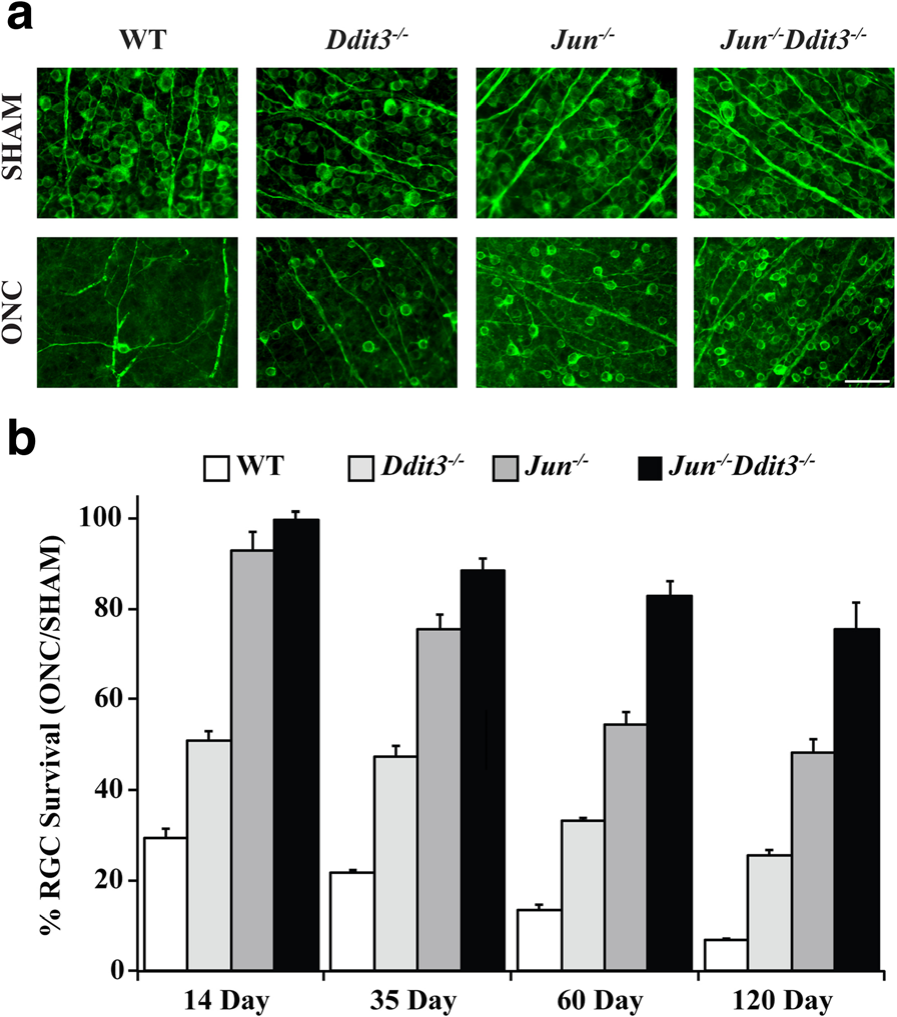
Combined *Jun/Ddit3* deficiency is more protective after axonal injury than either *Jun* or *Ddit3* deficiency alone. **a** Example of TUJ1 staining in control and experimental eyes at 120 days after ONC (scale bar = 50 μm). **b** *Ddit3, Jun,* and combined *Jun* and *Ddit3* deficient animals had significantly greater RGC survival than WT animals at all time points assessed (*p* < 0.001). Furthermore, there were significant differences (*P* < 0.001) found between the *Ddit3, Jun,* and combined *Jun* and *Ddit3* groups at all time points evaluated expect for between the *Jun,* and combined *Jun* and *Ddit3* 14 days after ONC. Data are plotted as the percentage survival relative to sham animals (*n* = 8 per condition per genotype for 14, 35, and 60 days after ONC, except *Jun* deficient ONC *n* = 7 at 35 days after ONC and *n* ≥ 6 per condition per genotype for 120 days after ONC; error bars represent SEM). Raw data in RGCs per mm^2^ and *P* values for all comparisons are presented in Table 1

Since our results suggest that *Jun* and *Ddit3* are independently upregulated after optic nerve injury, animals deficient in both *Jun* and *Ddit3* were generated to determine if the protection afforded by the individual deficiencies is additive. RGC survival was assessed 14, 35, 60, and 120 days after ONC with immunohistochemistry in retinal flat mounts using the RGC specific marker, TUJ1. Consistent with previous reports, deficiency of either *Jun* or *Ddit3* significantly increased RGC survival 14 and 35 days after ONC compared to WT animals [Fig. 3, Tables 1, 22, 33]. *Jun/Ddit3* deficient animals also had increased survival 14 and 35 days after ONC compared to WT animals (14 days after ONC- WT: 29% survival, *Ddit3*: 51%, *Jun*: 93%, *Jun/Ddit3*: 100%; 35 days after ONC- WT: 22%, *Ddit3*: 48%, *Jun*: 75%, *Jun/Ddit3*: 89%; *P* < 0.001 for all comparisons against WT animals) Furthermore, combined *Jun/Ddit3* deficient animals demonstrated increased survival compared to animals deficient in either *Jun* or *Ddit3* alone at 35 days (*p* < 0.001)*.* To determine if the protection afforded to RGCs by *Jun/Ddit3* deficiency was sustained, as in in *Bax* deficient mice [7, 50], a cohort of animals was examined 60 and 120 days after ONC. At both of these time points, RGC survival in *Jun, Ddit3,* and *Jun/Ddit3* deficient retinas was increased compared to WT retinas. RGC survival in combined *Jun/Ddit3* deficient mice was also significantly increased compared to single deletion of *Jun* or *Ddit3* in animals at both 60 days and 120 days after ONC (60 days after ONC- WT: 14 survival, *Ddit3*: 33%, *Jun*: 54%, *Jun/Ddit3*: 83%; 120 days after ONC- WT: 7%, *Ddit3*: 25%, *Jun*: 48%, *Jun/Ddit3*: 75%; *P* < 0.001 for all comparisons). The RGC protection conferred by single *Jun* deficiency and combined *Jun* and *Ddit3* deficiency may be even higher since ~22% of RGCs in *Jun* and *Jun/Ddit3* deficient retinas still express JUN due to incomplete recombination of the *Jun* floxed allele. Thus, the RGC protection afforded by combined *Jun*/*Ddit3* deficiency is sustained after axonal injury. These results demonstrate that JUN and DDIT3 act as the two principal signaling nodes through which all pro-apoptotic signaling converges resulting in RGC death after axonal injury.

**Table 1:** Combined *Jun/Ddit3* deficiency is more protective after axonal injury than *Jun* or *Ddit3* deficiency alone. (A) RGC per mm^2^ and (B) Percent survival was calculated for WT, *Ddit3* deficient, *Jun* deficient, and combined *Jun/Ddit3* deficient retinas at 14, 35, 60, and 120 days after ONC. Percent survival was defined as the mean number of RGCs after ONC divided by the mean number of RGCs after SHAM. Values are displayed plus/minus SEM. (C) One-way ANOVA followed by the Tukey’s post hoc test for multiple comparisons was used to analyze RGC cell counts at 14, 35, 60, and 120 days after ONC

A. RGC per mm^2^				
	WT	*Ddit3* ^*−/−*^	*Jun* ^*−/−*^	*Jun* ^*−/−*^ *Ddit3* ^*−/−*^
14 Day ONC	976.4 ± 59.1	1625.4 ± 62.8	2954.5 ± 128.5	3053.9 ± 48.6
14 Day SHAM	3306.8 ± 47.3	3191 ± 25.0	3178.5 ± 73.9	3060.6 ± 42.5
35 Day ONC	681.4 ± 92.5	1484.8 ± 68.7	2340.5 ± 98.8	2729.8 ± 83.9
35 Day SHAM	3149.4 ± 51.7	3125.8 ± 30.6	3103.5 ± 51.7	3085.4 ± 48.0
60 Day ONC	427.2 ± 79.7	1041.4 ± 14.0	1635.9 ± 79.7	2577.4 ± 100.4
60 Day SHAM	3166.7 ± 42.4	3122.9 ± 61.1	3006.3 ± 42.4	3108.2 ± 19.9
120 Day ONC	219.0 ± 8.3	797.0 ± 40.1	1498.0 ± 88.3	2328.0 ± 179.4
120 Day SHAM	3178.0 ± 30.1	3116.0 ± 52.4	3101.1 ± 38.9	3086.0 ± 61.1
B. % Survival (ONC/SHAM)				
	WT	*Ddit3* ^*−/−*^	*Jun* ^*−/−*^	*Jun* ^*−/−*^ *Ddit3* ^*−/−*^
14 Day	29.5 ± 1.8	50.9 ± 0.6	93.0 ± 1.0	99.8 ± 0.3
35 Day	21.6 ± 2.0	47.5 ± 2.2	75.4 ± 0.4	88.5 ± 1.3
60 Day	13.5 ± 4.0	33.3 ± 3.2	54.4 ± 2.6	82.9 ± 2.8
120 Day	6.9 ± 1.6	25.3 ± 2.7	48.3 ± 3.2	75.4 ± 5.8
C. One-way ANOVA followed by the Tukey’s post hoc test				
14 Days after ONC				
		WT	*Ddit3* ^*−/−*^	*Jun* ^*−/−*^
*Ddit3* ^*−/−*^		<0.0001	–	–
*Jun* ^*−/−*^		<0.0001	<0.0001	–
*Jun* ^*−/−*^ *Ddit3* ^*−/−*^		<0.0001	<0.0001	0.2107
35 Days after ONC				
		WT	*Ddit3* ^*−/−*^	*Jun* ^*−/−*^
*Ddit3* ^*−/−*^		<0.0001	–	–
*Jun* ^*−/−*^		<0.0001	<0.0001	–
*Jun* ^*−/−*^ *Ddit3* ^*−/−*^		<0.0001	<0.0001	0.0024
60 Days after ONC				
		WT	*Ddit3* ^*−/−*^	*Jun* ^*−/−*^
*Ddit3* ^*−/−*^		<0.0001	–	–
*Jun* ^*−/−*^		<0.0001	<0.0001	–
*Jun* ^*−/−*^ *Ddit3* ^*−/−*^		<0.0001	<0.0001	<0.0001
120 Days after ONC				
		WT	*Ddit3* ^*−/−*^	*Jun* ^*−/−*^
*Ddit3* ^*−/−*^		<0.0001	–	–
*Jun* ^*−/−*^		<0.0001	<0.0001	–
*Jun* ^*−/−*^ *Ddit3* ^*−/−*^		<0.0001	<0.0001	<0.0001

### *Dlk* is not the sole shared upstream regulator of *Jun* and *Ddit3* activity

**Fig. 4:**
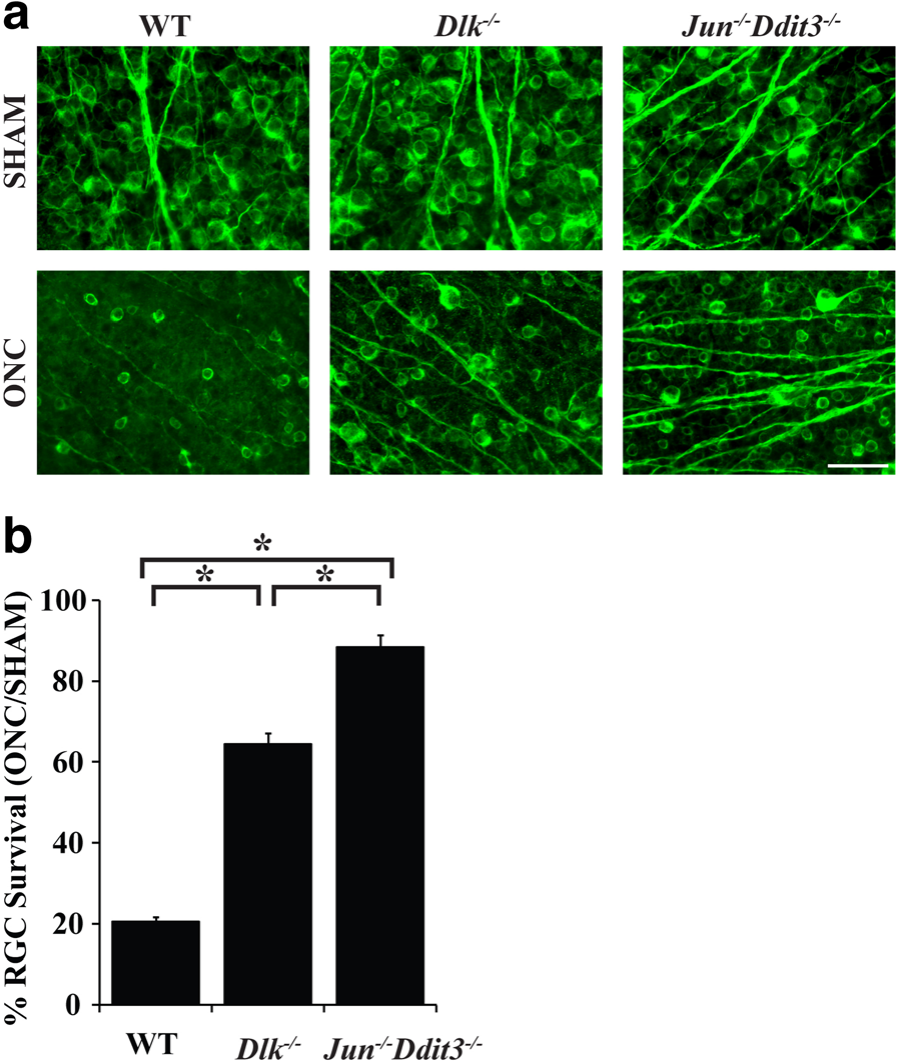
Combined *Jun/Ddit3* deficiency is more protective after axonal injury than *Dlk* deficiency. **a** Example images of TUJ1 staining in control and experimental eyes 35 days after ONC (scale bar = 50 μm). **b** TUJ1+ cell counts showed that *Dlk* deficient and combined *Jun/Ddit3* deficient animals had significantly greater RGC survival than WT mice 35 days after ONC (% given compared to control eyes of same genotype, WT: 20.4% survival, *Dlk*: 64.3% *Jun/Ddit3:* 88.5%; *, P < 0.001, n = 8 per condition per genotype except n = 7 for *Dlk* deficient animals; error bars represent SEM). Importantly, the protection provided by *Jun/Ddit3* deficiency was significantly greater than the protection provided by *Dlk* deficiency alone (P < 0.001)

Dual leucine kinase (DLK) is a mitogen-activated protein kinase kinase kinase (MAP3K) and a critical regulator of RGC death after optic nerve injury [35, 36, 43]. Pharmacologic inhibition of DLK has been shown to be protective in rodent models of ocular hypertension suggesting the importance of this molecule in the pathogenesis of glaucoma [36]. After axonal injury, DLK is a major activator of JNK signaling and regulates both apoptosis and Wallerian degeneration. After optic nerve injury, *Dlk* deficiency decreases the somal pool of JNK, attenuates somal JUN accumulation, and ultimately increases survival of RGCs as compared to WT animals [35, 36, 43]. Though protective of RGC somas, *Dlk* deficiency does not prevent activation of axonal JNK [43]. *Dlk* has also been proposed to regulate *Ddit3* expression after axonal injury. Unbiased gene expression profiling after mechanical axonal injury demonstrated that *Dlk* deficiency significantly reduced the expression of DDIT3 [35]. Together these results raise the possibility that DLK could be upstream of both *Jun* and *Ddit3,* thus making *Dlk* an important common upstream regulator of RGC death after axonal injury. To test this hypothesis, RGC survival was directly compared in WT, *Dlk,* and *Jun/Ddit3* deficient retinas 35 days after ONC. This time point was chosen because there was significant difference in RGC loss between WT, *Jun*, *Ddit3* and *Jun/Ddit3* deficiency mice after ONC. TUJ1+ cell counts confirmed that both *Dlk* and *Jun/Ddit3* deficiency provide significant protection to RGCs as compared to WT animals (Fig. 4; *P* < 0.001 *n* ≥ 7 per group; WT: 20.4% survival, *Dlk*: 64.3%, *Jun/Ddit3*: 88.5%). Furthermore, there was greater protection in *Jun/Ddit3* deficient retinas compared to *Dlk* deficient retinas (*p* < 0.001) suggesting *Dlk* may not be the sole upstream regulator of JUN and DDIT3 activation.

### Neither *Jun*, *Ddit3*, nor combined *Jun* and *Ddit3* deficiency alters axonal degeneration after mechanical optic nerve injury

**Fig. 5:**
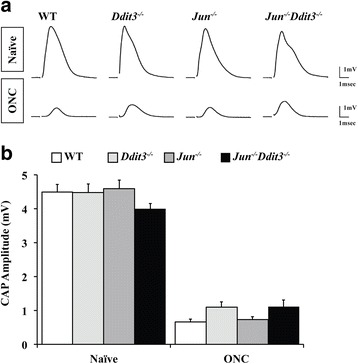
Neither *Jun*, *Ddit3*, or combined *Jun*/*Ddit3* deficiency alters axonal degeneration after mechanical optic nerve injury. To assess the role of JUN and DDIT3 in axonal degeneration, compound action potentials (CAPs) were recorded from WT, *Jun, Ddit3*, and combined *Jun*/*Ddit3* animals 5 days after ONC, a time point when there is significant loss of CAP amplitudes in WT mice [43]. **a** Representative traces show that sham eyes from all genotypes had normal action potentials, while amplitudes were reduced about 80% in all cases after ONC. **b** Quantification of CAPs from WT, *Jun, Ddit3*, and combined *Jun*/*Ddit3* animals showed that there were no differences among the CAP amplitudes of naïve, uninjured eyes of all genotypes (*P* > 0.05). All genotypes had significantly reduced amplitudes after ONC as compared to naïve animals (P < 0.001 for all comparisons). CAP amplitudes of *Jun, Ddit3*, and combined *Jun*/*Ddit3* animals were not significantly different after ONC from those of control animals (P > 0.05, *n* = 4 for each genotype and condition)

RGC injury and axonal degeneration are important components of glaucomatous neurodegeneration [2, 8, 9]. After axonal injury, there are differential responses within the RGC somal and axonal compartments [51, 52]. There is spatial compartmentalization of molecular mechanisms that control RGC somal (cell body) and RGC axonal degeneration. Thus, it is necessary to test the role of JUN and DDIT3 in both somal death and axonal degeneration. DDIT3 deficiency promotes optic nerve survival in models of glaucoma, including mechanical optic nerve injury and increased intraocular pressure [33, 34, 37]. *Jun* deficiency has also been shown to protect axons from degeneration in sensory neuron culture [53]. In the DBA/2 J ocular hypertensive mouse model of glaucoma, *Jun* deficiency protected RGC somas but not axons from glaucomatous injury [54]. Thus, JUN does not appear to be required for axonal degeneration in adult RGCs after a glaucomatous injury. To test the hypothesis that single and/or combined deficiency of *Jun* and *Ddit3* may protect against axonal degeneration, optic nerve function was tested after mechanical optic nerve injury. Compound action potentials (CAP) were measured in WT, *Jun*, *Ddit3*, and *Jun/Ddit3* deficient mice five days after ONC, a time when WT animals demonstrate significant reduction in CAP amplitudes [43]. No differences in amplitudes were observed across the four groups, suggesting that deficiency of *Jun* and *Ddit3,* either singly or together, does not prevent for axonal degeneration after mechanical optic nerve injury (Fig. 5; *P* > 0.05 for all comparisons; *n* = 4 for all groups).

## Discussion

Axonal injury is a critical component in many neurodegenerative diseases including glaucomatous neurodegeneration [2, 8, 9, 13, 14, 55, 56]. After axonal injury, RGCs undergo apoptosis in a BAX-dependent manner [5–7]. The molecular pathway that leads from axonal disruption to BAX activation is only partly defined. In fact, no molecule other than BAX is known to be required for BAX dependent RGC death after axonal injury. The transcription factors JUN and DDIT3 are expressed in RGCs after axonal injury [22–26, 31–34, 57, 58]. Both JUN and DDIT3 are known to have pro-death functions in neurons [42, 59, 60]. In RGCs, individual deficiency of *Jun* and *Ddit3* provides significant, but incomplete protection of RGCs after axonal injury [22, 23, 33]. Here, we sought to determine if these pathways are independent of each other and if so, whether combined deficiency of JUN and DDIT3 provides complete protection to RGCs after ONC. JUN and DDIT3 appear to control RGC death independently. Single deficiency of *Jun* or *Ddit3* significantly protected RGCs somas at all time points tested. Combined deficiency of *Jun* and *Ddit3* provided robust protection of RGCs after axonal injury, e.g., 89% survival 35 days after ONC. Furthermore, accounting for the incomplete deletion of *Jun* in RGCs, *Jun/Ddit3* deficiency may protect over 95% of RGCs 120 days after ONC. Thus, combined deficiency of *Jun* and *Ddit3* appears to provide nearly complete protection when accounting for incomplete recombination of the *Jun* allele. Together, JUN and DDIT3 activity accounts for the vast majority of apoptotic signaling in RGCs after axonal injury. Furthermore, because JUN and DDIT3 canonically function as transcription factors, it appears that after axonal injury RGC death requires transcriptional events.

JUN and DDIT3 activation may function as key molecular hubs that integrate upstream signaling events triggered by glaucomatous axonal injury. Given that the canonical function of JUN and DDIT3 is to regulate transcription, their activation likely leads to the transcription of pro-death genes that ultimately trigger RGC apoptosis. The MAPK and ER stress pathways have both been suggested as pharmacologic targets for glaucomatous injury and there is also some evidence for physiologic overlap [35, 40, 49, 61]. Both systems are known to control pro-death genes in neurons. In RGCs, JUN directly controls the pro-death targets BIM, ATF3, and HRK [23, 46]. In other cell types, DDIT3 has been shown to control pro-death components including DR5 (death receptor-5 or TNFRSF10B), BIM, and GSN [39, 62, 63]. JUN, in RGCs, and DDIT3, in other cell types, have also been shown to inhibit pro-survival targets, such as BCL-2 [23, 64].

Interestingly, JUN and DDIT3 may have overlapping functions. DDIT3 can form protein-protein complexes with JUN and other members of the activator protein-1 (AP-1) transcription factor family [61, 62]. Given these interactions and the fact that activation of both *Jun* and *Ddit3* signaling cascades may culminate in BAX-dependent apoptosis, it is possible that together JUN and DDIT3 may co-regulate downstream effector targets in RGCs after axonal injury. Further work using transcriptomics will be needed to define the downstream effector molecules differentially and mutually controlled by *Jun* and *Ddit3.* In addition to glaucoma-relevant injuries, JUN and DDIT3 have been implicated in other neurodegenerative diseases [42, 60]. Thus, investigation into the downstream molecular targets of JUN and DDIT3 may determine molecular events broadly important for neurodegeneration.

Identification of the upstream regulator(s) of *Jun* and *Ddit3* will be an important step towards defining the critical pathway that leads from axonal injury to RGC degeneration. Potential molecules that have been suggested to activate both MAPK and endoplasmic reticulum stress signaling in other systems include mTOR, TLR4, and ETN1 [65–70]. Further study, however, should be done to identify molecules that activate both JUN and DDIT3 signaling in RGCs. Identified molecules should then be carefully tested in a model of optic nerve injury or preferably, a model of ocular hypertension to determine 1) if these molecules are expressed at a time point consistent with glaucomatous injury 2) if manipulation of these molecules decreases JUN and DDIT3 expression and 3) if deficiency of these molecules protects RGC somas and their axons. These experiments might also reveal early molecular signaling events that occur in axons after a glaucomatous injury. *Dlk* is a MAP3K known to activate JNK-JUN signaling in RGCs and has also been proposed to regulate DDIT3 activation in RGCs [35, 43]. *Dlk* deficiency provided significant protection to RGCs after axonal injury. The level of protection afforded by *Dlk* deficiency was similar to that provided by *Jun* deficiency and about 50% more protection then provided by *Ddit3* deficiency. Like *Jun* deficiency, protection provided by *Dlk* deficiency was not complete. Combined *Jun/Ddit3* deficiency provided greater protection to RGCs than *Dlk* deficiency. Thus, *Dlk* is likely not the sole or only upstream regulator of JUN and DDIT3 in RGCs after axonal injury. While signaling upstream of JUN and DDIT3 may converge, it is also possible that there is not a sole common upstream molecular regulator of all JUN and DDIT3 signaling. The inciting injury in glaucomatous neurodegeneration itself or early sequelae of this perturbation may activate both *Jun* and *Ddit3* through separate mechanisms*.* Evidence has implicated several distinct cellular events induced by ocular hypertension-induced axonal injury, including: loss of trophic support, noxious glial signaling, neurovascular unit dysfunction, and disruption of the axonal cytoskeleton [16, 20, 71, 72]. Future work to determine a shared upstream regulator of *Jun* and *Ddit3* activation will be important for further defining the major molecular signaling cascades driving RGC degeneration. Regardless of whether the inciting injury in glaucomatous neurodegeneration is extrinsic or intrinsic to RGCs, sequentially stepping upstream of JUN and DDIT3 activation may define the earliest events in glaucoma.

Determining the events leading to MAPK and ER stress activation may be challenging because the MAPK and ER stress signaling pathways are complex. Both routes have multiple parallel pathways with both divergent and convergent elements [59, 73–75]. ER stress is the disruption of normal ER function and activation of the unfolded protein response (UPR) due to the accumulation of unfolded proteins and interruption of normal calcium regulation [76]. The three ER stress response proteins are inositol-requiring transmembrane kinase (IRE1), activating transcription factor-6 (ATF6), and protein kinase RNA-like endoplasmic reticulum kinase (PERK). DDIT3 may be a target of all three protein pathways, but is predominately activated by PERK signaling [77]. Previous work has demonstrated that IRE and ATF6 signaling (the largely pro-survival signals of ER stress) are diminished with persistent ER stress signals, while PERK signaling (largely pro-apoptotic) is maintained [78]. In the same study, overexpression of IRE1, led to enhanced cellular viability after axonal injury. XBP1, a component of the IRE arm, was also activated in RGCs after axonal injury [33, 37], while XBP1 and DDIT3 were differentially expressed [33, 37]. While DDIT3 expression was robust and sustained after axotomy, XBP1 was transiently expressed at modest levels early after injury [33]. Unlike deficiency of *Ddit3*, deficiency of *Xbp1* did not prevent RGC loss after axonal injury suggesting that *Ddit3* dependent signaling was the critical part of the ER stress response for RGC death.

Glaucoma is a chronic, age-related disease which may play out over years and potentially decades [3]. Given the extended time period of glaucomatous injury, chronic ER stress may lead to prolonged activation of PERK-DDIT3 signaling which may override the protective components of ER stress signaling. Furthermore, the unfolded protein response, primarily through the IRE1 arm, has also been shown to initiate phosphorylation of c-Jun N-terminal kinase (JNK) that in turn activates JUN [40–42]. Therefore, in addition to prolonged pro-apoptotic ER stress signaling, activation of ER stress signaling in glaucoma may lead to sustained pro-apoptotic JNK-JUN signaling.

Deficiency in *Jun*, *Ddit3*, or combined *Jun*/*Ddit3* did not prevent loss of axonal function as measured by compound action potential after mechanical axonal injury. It is possible that deficiency in these molecules could lessen morphological degeneration, which was not measured here. *Dlk* deficiency, an upstream regulator of JUN in RGCs, and *Ddit3* deficiency both provided some protection to RGC somas and axons in an acute intraocular pressure elevation mouse model of glaucoma [34, 36] Recently, we showed deficiency in *Jun* does not prevent axonal degeneration in a chronic ocular hypertensive mouse model of glaucoma, though it does provide some protection to RGC somas [54]. It is possible, however, that deficiency in both *Jun* and *Ddit3* will protect RGCs in a chronic, ocular hypertensive model of glaucoma. Interestingly, if combined deficiency of *Jun* and *Ddit3* protects both RGC axons and somas in a glaucoma model it will point to new transcriptional pathways underlying RGC degeneration in glaucoma. This protection would suggest that the transcription events that occur during IOP elevation are critical for axonal degeneration. Such events may not be identified after an acute mechanical axonal injury because of axonal severing. Therefore, it will be important to test the role of *Jun* and *Ddit3* in a chronic, age-related model of ocular hypertension, the DBA/2 J mouse, to determine if *Jun* and *Ddit3* are critical for glaucomatous ocular hypertensive injury.

## Conclusions

*Jun* and *Ddit3* are independently controlled transcription factors that together provide robust, near complete protection of RGCs after axonal injury. *Jun* and *Ddit3* are ideally positioned to integrate cell-signaling cascades after axonal injury with transcriptional regulation of RGC death. *Jun* and *Ddit3* signaling have been shown to be involved in other injuries to RGCs including ischemic injury, diabetic retinopathy, and traumatic optic neuropathy (and traumatic brain injury) [23, 27, 33, 58, 79–82]. Given the robust protection shown here after RGC injury, it will be interesting to determine if dual activation of *Jun* and *Ddit* may define a canonical signaling pathway (culminating in BAX activation) for RGC death. Furthermore, it will be important to test the effectiveness of inhibiting both JNK-JUN and DDIT3-dependent ER stress signaling in other injury paradigms that result in RGC death. As axonal injury is a critical event in many different diseases, it is possible that JUN and DDIT3 may control neural degeneration after axonal injury in other neuron types. Thus, the finding that together JUN and DDIT3 control RGC death after axonal injury may be broadly applicable to understanding the molecular signaling pathways that control neurodegeneration.

## Abbreviations

ACSF: artificial cerebrospinal fluid
AP-1: activator protein 1
ATF6: activating transcription factor 6
CAP: compound action potential
CHOP: CCAAT/enhancer binding homologous protein
DDIT3: DNA damage inducible transcript 3
DLK: dual leucine kinase
DR5: death receptor 5
ER: endoplasmic reticulum
IRE1: inositol-requiring transmembrane kinase 1
JNK: c-Jun N-terminal kinase
MAP3K: mitogen-activated protein kinase kinase kinase
MAPK: mitogen-activated protein kinase
ONC: optic nerve crush
PERK: protein kinase RNA-like endoplasmic reticulum kinase
PFA: paraformaldehyde
RGC: retinal ganglion cell
TUJ1: βIII tubulin
UPR: unfolded protein response
WT: wildtype
